# Bioinformatics-based prediction of conformational epitopes for Enterovirus A71 and Coxsackievirus A16

**DOI:** 10.1038/s41598-021-84891-6

**Published:** 2021-03-11

**Authors:** Liping Wang, Miao Zhu, Yulu Fang, Hao Rong, Liuying Gao, Qi Liao, Lina Zhang, Changzheng Dong

**Affiliations:** 1 Department of Preventive Medicine, Zhejiang Provincial Key Laboratory of Pathological and Physiological Technology, School of Medicine, Ningbo University, Ningbo, China; 2 Department of Infection Control, Heze Municipal Hospital, Shandong, China; 3 The Affiliated People’s Hospital of Ningbo University, Ningbo, China

**Keywords:** Computational biology and bioinformatics, Vaccines, Virology

## Abstract

Enterovirus A71 (EV-A71), Coxsackievirus A16 (CV-A16) and CV-A10 are the major causative agents of hand, foot and mouth disease (HFMD). The conformational epitopes play a vital role in monitoring the antigenic evolution, predicting dominant strains and preparing vaccines. In this study, we employed a Bioinformatics-based algorithm to predict the conformational epitopes of EV-A71 and CV-A16 and compared with that of CV-A10. Prediction results revealed that the distribution patterns of conformational epitopes of EV-A71 and CV-A16 were similar to that of CV-A10 and their epitopes likewise consisted of three sites: site 1 (on the “north rim” of the canyon around the fivefold vertex), site 2 (on the “puff”) and site 3 (one part was in the “knob” and the other was near the threefold vertex). The reported epitopes highly overlapped with our predicted epitopes indicating the predicted results were reliable. These data suggested that three-site distribution pattern may be the basic distribution role of epitopes on the enteroviruses capsids. Our prediction results of EV-A71 and CV-A16 can provide essential information for monitoring the antigenic evolution of enterovirus.

## Introduction

Hand, foot and mouth disease (HFMD) is a common febrile disease caused by human enteroviruses, which mainly inflicts infants and young children, resulting in the appearance of vesicular rashes on hands, feet and buccal mucosa^1^. Since 1997, numerous great outbreaks of HFMD have occurred in the Asia–Pacific region involving Malaysia, Australia, Taiwan and mainland China^2–8^. Thereafter, HFMD was listed under the surveillance by Chinese Center for Disease Control and Prevention. From 2008 to 2015, a total of more than 13 million HFMD cases (probable and laboratory-confirmed) were reported, among which the severe and fatal cases were more than 120,000 and 3000, respectively^9^. Enterovirus A71 (EV-A71), Coxsackievirus A16 (CV-A16), CV-A10 and CV-A6, members of the enterovirus species A (EV-A), are major causative agents of HFMD. CV-A16-induced HFMD is often benign and mild, while EV-A71-associated infections involve mild and severe cases manifesting neurological complications, and even death^10–13^.

**Figure 1:**
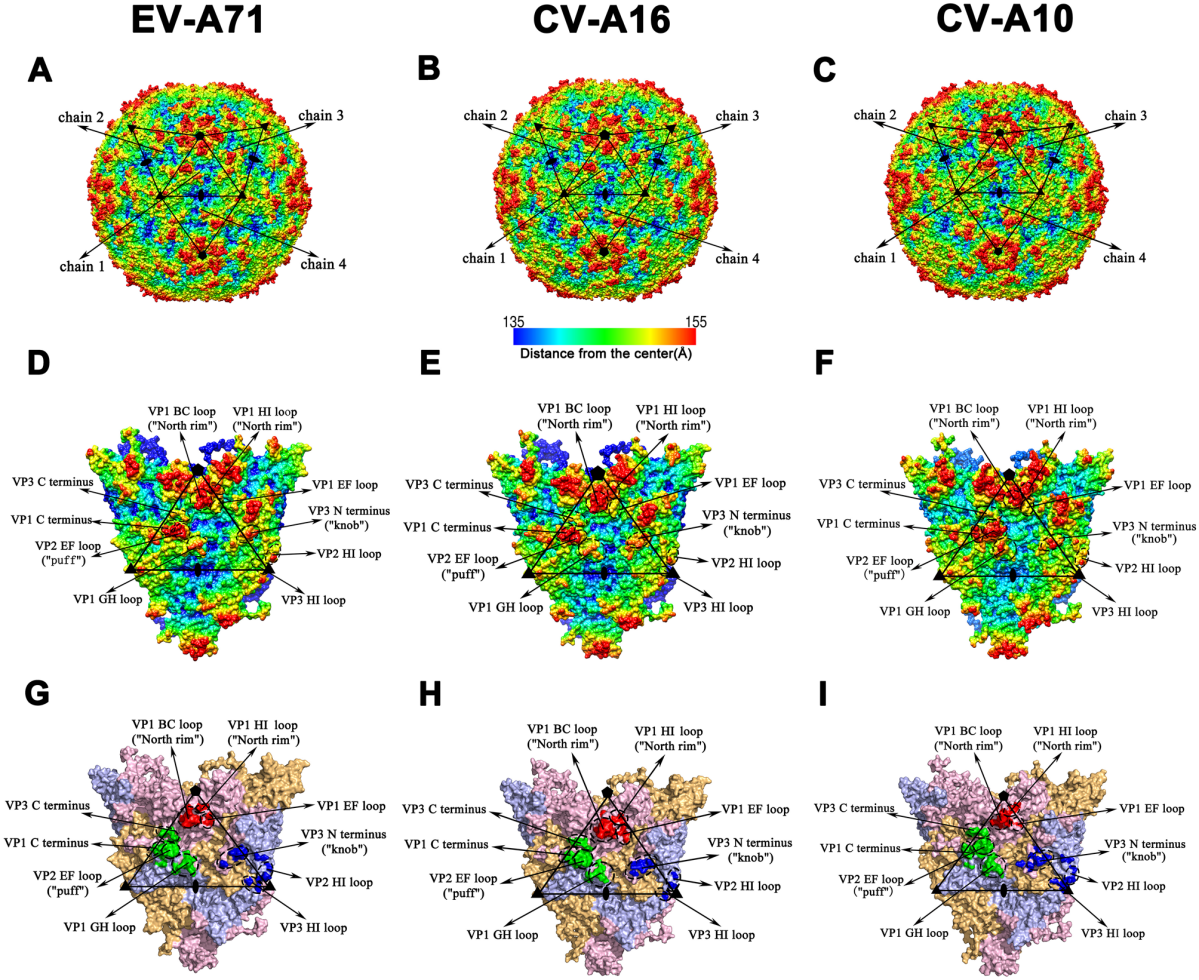
Capsid structures of EV-A71, CV-A16 and CV-A10. (**A–C**) Radius-colored surface representations of EV-A71 (**A**), CV-A16 (**B**) and CV-A10 (**C**). Capsids are colored according to their radius from blue to red as shown in the color bar. The multiple chains consisting of four units labeled as chain 1–4 are indicated by black triangles. Fivefold, threefold and twofold vertexes are marked as pentagons, triangles and ovals, respectively. (**D–F**) Zoom-in views of multiple chains and capsid characteristics of EV-A71 (**D**), CV-A16 (**E**) and CV-A10 (**F**). The color scheme is as described in the legend to (**A–C**). The central chain (chain 1) is outlined by a black triangle. (**G–I**) The predicted conformational epitopes on the chain 1 of EV-A71 (**G**), CV-A16 (**H**) and CV-A10 (**I**). The prediction results of conformational epitopes for CV-A10 are obtained from our previous work with permission from the journal^44^. Structural proteins VP1-3 are shown as surface representations in light pink, light blue and light orange, respectively. The chain 1 is outlined as the (**D**–**F**). The conformational epitopes clustered into three sites are highlighted in red (site 1), green (site 2) and blue (site 3), respectively.

EV-A, Poliovirus (PV) and Rhinovirus (RV), belonging to the *enterovirus* genus within the family *Picornaviridae*, have similar genome and viral structure^14,15^. The genome of EV-A71 is a positive-sense single-strand RNA generating a large polyprotein that is subsequently cleaved into three precursor proteins, termed as P1, P2, and P3. P1 encodes four viral proteins (VPs) including VP1 to VP4, while P2 and P3 encode seven non-structural proteins^16,17^. The mature viral capsid is icosahedral containing 60 copies of the protomer composed of four proteins, VP1-4. Of these, VP1-3 together forms the shell of the virion whereas VP4 is linked to viral RNA and entirely internal to the capsid. It is believed that neutralization epitopes resided mainly in VP1-3, but only few in VP4 (Fig. 1). The VP1-3 all have a basically similar structure consisting of an eight-stranded β-barrel called β-sandwich “jelly-roll” folds or immunoglobulin-like folds which is linked by loops of varying lengths such as BC and CD loops. A deep surface depression “canyon” encircling the icosahedral fivefold vertex is possibly responsible for receptor binding (Fig. 1). One rim (“north rim”) of this canyon is mainly composed of VP1 and the other (“south rim”) is formed by VP2 and VP3, which can be engaged with antibodies to block receptors binding to the virus^16,17^.

A number of vaccines against EV-A71 in China have made significant achievements in clinical trials and three of them have been successfully licensed by National Medical Products Administration of China (https://www.nmpa.gov.cn) in 2015 and 2016^18–22^. As the only vaccines against HFMD in the world, three inactivated monovalent vaccines against EV-A71 play an active role in health protection of children. However, it has to be noticed that these vaccines are incapable of inducing sufficient cross-protection against other enteroviruses such as CV-A16 and CV-A10^23^. This means that they can only provide protection against EV-A71-associated HFMD and be futile to other enteroviruses infections. Moreover, since EV-A71 is constantly mutating and evolving, the protective effect of vaccines will gradually decline. Thus, it is necessary to supervise the antigenic evolution of EV-A71 and update the vaccine in time like influenza viruses. Based on the antigenic epitopes of influenza virus, many models or algorithms had been successfully developed to monitor the evolution of its antigenicity, predict dominant strains and prepare vaccines^24–28^.

Antigenic epitopes, also called antigenic determinant, are divided into two categories: linear (or continuous) epitopes and conformational (or discontinuous) epitopes. The linear epitopes are formed by continuous amino acid residues, while the conformational epitopes consist of residues that are distantly separated in the sequence but spatially proximal^15,29^. The conformational epitopes can specifically bind to antibodies that block the cellular receptors binding to virus; furthermore, they are the basis of viral antigenicity and used to distinguish the serotypes of enteroviruses^15,29^. Structural biology methods such as cryo-electron microscopy (Cryo-EM) and X-ray crystallography were used to determine the three-dimensional (3D) structure of the virus-Fab complexes and recognize the binding region (conformational epitope or called “footprint”) of Fab with EV-A71^30–35^ or CV-A16^36^.

As a powerful approach for epitopes determination, Cryo-EM is expensive, time-consuming and labor-intensive^37^. So far, only a few conformational epitopes of enteroviruses have been reported and mainly focused on EV-A71 or PV1^38,39^. The Bioinformatics prediction algorithm, as a supplement method, can quickly and systematically predict the viral epitopes and screen alternative epitopes for experimental research. The basic principle of the algorithm is to take the 3D structure of a protein antigen as a spherical or ellipsoidal shape and search for the sites extremely exposed or easily binding to antibodies^40–42^. For example, DiscoTope integrates the propensity scores into an average surface neighborhood attribute and regards discontinuous amino acid residues with high propensity score as conformational epitopes^40^. However, presently the majority of predicted algorithms for conformational epitopes are not based on the whole viral capsid proteins but a single protein such as VP1. These algorithms do not take into account the structural features of the viral capsid; therefore, the reliability of the prediction for enterovirus is very low. Borley et al. creatively proposed the multiple chains algorithm to predict the conformational epitopes of foot and mouth disease virus (FMDV)^43^. The algorithm was proved highly reliable in theory and practice. Both human enteroviruses and FMDV belong to the family *Picornaviridae* and share a similar viral capsid structure. Hence, the multiple chains algorithm developed by Borley et al. can also be competent in prediction for conformational epitopes of human enteroviruses. Based on the multiple chains algorithm, we developed a conformational epitope prediction algorithm for CV-A10 with high prediction reliability, and found that the conformational epitopes of CV-A10 were clustered into three sites (site 1, site 2 and site 3)^44^.

In this study, the conformational epitopes of EV-A71 and CV-A16 were systematically predicted with a similar algorithm like before and compared with that of CV-A10. The structural differences of the conformational epitopes (including the primary, secondary, tertiary and capsid structures) among EV-A71, CV-A16 and CV-A10 were explored.

## Results

### Prediction results of the conformational epitopes of EV-A71 and CV-A16

The predicted conformational epitopes were shown in Table 1 and Fig. 1G,H. Like CV-A10, the conformational epitopes of EV-A71 and CV-A16 were clustered into three sites (site 1, site 2 and site 3), one of which was composed of two or three parts. For instance, site 1 consisted of site 1a, site 1b and site 1c; site 2 was comprised of site 2a, site 2b and site 2c and site 3 was composed of site 3a and site 3b. The majority of epitopes were located on the rims of canyon, and the remaining epitopes were close to the threefold vertex (Fig. 1G–I). In detail, site 1, which contained VP1 BC, EF and HI loops, was located on the north rim around the fivefold vertex. Both site 2 and site 3a stood on the south rim, and the former was on the puff and the latter in the knob. Site 2 was composed of VP1 GH loop, VP2 EF loop, VP1 C terminus and VP3 C terminus; site 3a was composed of individual residues in VP1 C terminus and VP3 N terminus; site 3b near the threefold vertex consisted of some residues in VP2 BC, HI and VP3 HI loops (Table 1, Fig. 1G–I). Notably, the discontinuous amino residues in the sequence could form the same epitope. For example, residues from VP1 BC, EF and HI loops together formed the site 1 and some residues from VP1-3 corporately formed the site 2 and site 3. Additionally, not all the residues in three sites came from the same chain (Supplementary Fig. S1). In fact, a few residues in site 2c, site 3a and site 3b were from the neighbouring chains, and the residues from two different chains worked together as a common epitope. Site 2 was not only adjacent to the neighboring site 1 at the narrowest of canyon but also near the neighboring site 3a, which suggested that it was possible for them to interact with the same monoclonal antibody (mAb) or receptor. All of these fully reflected the characteristics of conformational epitopes that discontinuous residues in the sequence could be spatially adjacent and constitute the same epitope.

**Table 1:** Prediction results of conformational epitopes of EV-A71, CV-A16 and CV-A10.

Name of epitopes	Capsid characteristics	Name of sub-epitopes	Secondary structure	EV-A71^a^	CV-A16	CV-A10^b^
site 1	north rim	site 1a	VP1 BC loop	94D, **96P**, 97L, 98K, **99G**, **100T**, 101Q, **102N**, 103P, 104N	94T, **96P**, 97T, 98T, **99G**, **100T**, 101Q, **102**N, 103T, 104D, 106Y, 107V	94N, 96T, 97D, 98G, 99G, 100T, 101D, 102T, 103T, 106V
		site 1b	VP1 EF loop	**165S**, 167E, **168S**	**165S**, 167D, **168S**, 169F	164G, 167A
		site 1c	VP1 HI loop	241S, **242K**	240I, 241E, **242K,** 245H	240E, 241A, 244L
site 2	puff	site 2a	VP1 GH loop	**213E**, **214H**, 215K, **216Q**, 217E, 218K, **220L**	**213E**, **214H**, 215L, **216Q**, 217A, 218N, **220L**	212Q, 213H, 214P, 215E, 216T, 217S, 219T
		site 2b	VP1 C terminus	**272L**, **273F**, 280A, **281G**, **282N**, 283S	**272L**, **273F**, 280K, **281G**, **282N**, 283D	271L, 272L, 279D, 280S, 281S, 282K
			VP2 EF loop	135V, **136A, 137G**, **138G**, **139T**, **140G**, 141T, **142E**, 143D, 144T, **147P,** 149K, 161Q	135I, **136A, 137G**, **138G**, **139T**, **140G**, 141N, **142E**, 143N, 144S, **147P,** 149A	136G, 137S, 138N, 139T, 140K, 141P, 142N, 143E, 144A,145P, 148G, 150T, 162H
		site 2c	VP3 C terminus	236L, **237Q**, **238T**, 239G, 240T, **242Q**	236E, **237Q**, **238T**, 239A, 240N, **242Q**	234T, 235Q, 236Q, 237A, 238V, 240Q
site 3	knob	site 3a	VP1 C terminus	292T	290S, 292D	285N, 289D, 291S, 292S, 297N
			VP3 N terminus	59P, **60T**, 61N, 62A, **63T,** 64S	59K, **60T**, 62E, **63T,** 64T	58T, 59T, 60E, 61A, 62T
	threefold vertex	site 3b	VP2 BC loop	72E, 74S	–	72Q, 74N
			VP2 HI loop	225D, 226Q, **227G**, 230P	226T, **227G**, 230S	226S, 227S, 228G, 229A, 231T
			VP3 HI loop	**206I**, **207G**, 210N	**206I**, **207G**	204P, 205E, 206T, 208G

^a^The bold letters represent the conserved amino acids between EV-A71 and CV-A16. The underlines represent the conserved amino acids among EV-A71, CV-A16 and CV-A10.

^b^The prediction results of conformational epitopes of CV-A10 are from our previous study with the permission of the journal^44^.

**Figure 2:**
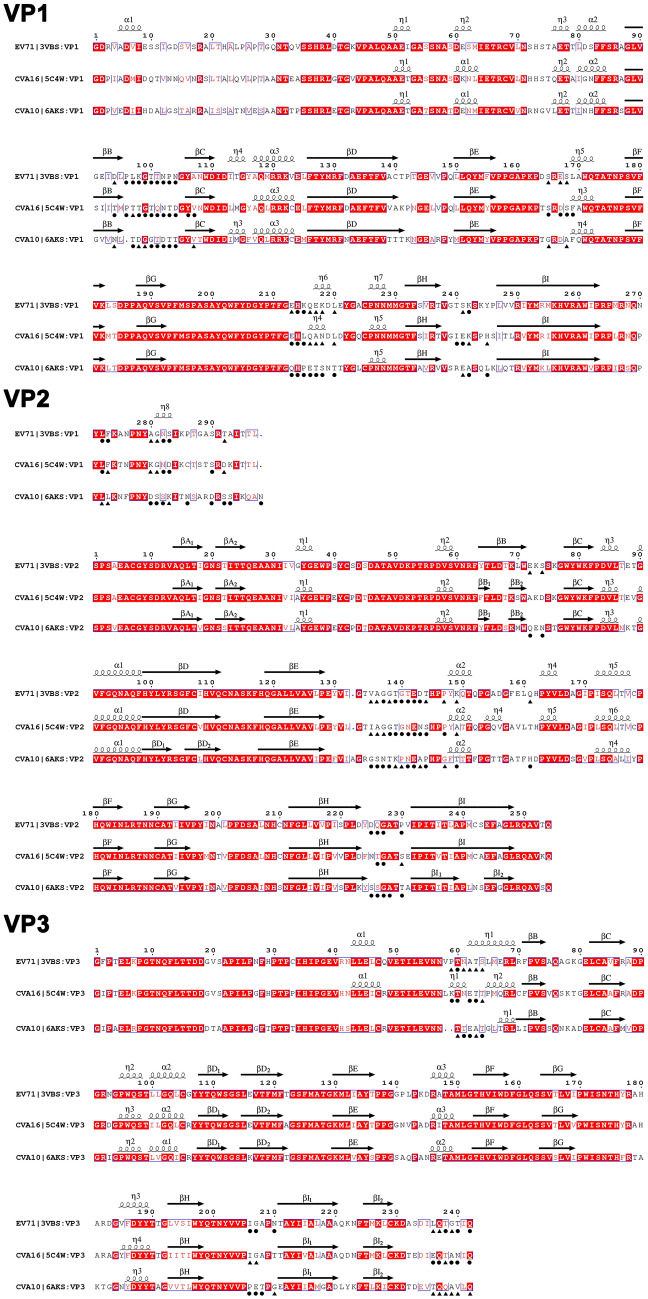
Sequence alignments of three VPs (VP1, VP2 and VP3) of EV-A71, CV-A16 and CV-A10. ESPript representation of structure-based sequence alignments of EV-A71 (GenBank accession no: EU703812), CV-A16 (GenBank accession no: JQ354992) and CV-A10 (GenBank accession no: KP009574) are shown. The second structure elements (β-sheets and α-helices) are shown above the corresponding alignment as arrows (β-sheets) and spirals (α-helices), respectively. The predicted epitopes are shown as black circles (core epitopes) and triangles (surrounding epitopes). The predicted results of CV-A10 are from our previous work. The alignments are colored according to the sequence identity among EV-A71, CV-A16 and CV-A10, on a scale of white (no identity) to red (full identity).

The sequence alignments of EV-A71 and CV-A16 were shown in Fig. 2. The full length of VP1-3 of EV-A71 and CV-A16 was 793 amino acid residues. And the three VPs of these two serotypes were exactly the same length, with 297 (VP1), 254 (VP2) and 242 (VP3) residues. The sequence identities of VP1, VP2 and VP3 were 71.4%, 84.3% and 83.5%, respectively, whereas those of the corresponding epitope sequences were only 48.6%, 42.1% and 53.3%. These data indicated that the epitopes were mainly located in highly variable regions. Totally, there were 63 residues of EV-A71 and CV-A16 predicted as epitopes, of which 57 residue positions were shared by two serotypes accounting for 90.5% of the total positions (Table 1, Fig. 2). This result illustrated the positions of epitopes were highly consistent between EV-A71 and CV-A16. At the shared positions, 30 (52.6%) residues were conserved indicating that variation of epitope residues caused the antigenic difference between these two serotypes. For example, there were 10 conserved residues 94D, 96P, 97L, 98K, 99G, 100T, 101T, 102N, 103P and 104N in the VP1 BC loop of EV-A71, by contrast, 10 residues 94T, 96P, 97T, 98T, 99G, 100T, 101Q, 102N, 103T and 104D of CV-A16 were conserved at the same positions as EV-A71. In addition, 29 (VP1), 19 (VP2) and 15 (VP3) residues of EV-A71 and 35 (VP1), 15 (VP2) and 13 (VP3) residues of CV-A16 were predicted as conformational epitopes, which proved that VP1 was the structural protein that most easily formed epitopes. In line with this, the sequence identity (71.4%) of VP1 between EV-A71 and CV-A16 was the lowest and the length of VP1 was the longest among all VPs. Unexpectedly, although the sequence identities of VP2 (84.3%) and VP3 (83.5%) were very high, their epitope sequence identities (VP2: 42.1%, VP3: 53.3%) were much lower. This prompted that VP2 and VP3, constituting the site 2 and site 3, played an essential role in the antigenic evolution. The sequence identities of VP1-3 of CV-A10 with EV-A71, CV-A10 with CV-A16 were 66.9% and 68.7%, respectively, which was proximately 10% lower than that (79.3%) between EV-A71 and CV-A16 (Fig. 2). However, the identities of epitope sequence of CV-A10 with EV-A71, CV-A10 with CV-A16 were only 14.1% and 24.3%, respectively, which were much lower than that of EV-A71 with CV-A16 (47.8%). The same situation could be seen in Table 1. Amongst all the epitopes of CV-A10, only nine residues were conserved with EV-A71 and sixteen residues with CV-A16. Furthermore, only eight residues were conserved among these three distinguished serotype viruses. All of above implied that more differences existed between epitopes of CV-A10 with EV-A71 and CV-A16.

EV-A71 and CV-A16 had highly consistent secondary structures (Fig. 2). The lengths and locations of eight β sheets in three VPs were almost identical. Likewise, the lengths of loops, N terminuses and C terminuses were identical as well. Loops, exposed on the outer surface of capsid, were highly variable secondary structures and prone to form epitopes. About two-thirds of epitopes of EV-A71 and CV-A16 were located in the loops (Table 1, Fig. 2). For example, site 1a, site 1b and site 1c were located in the VP1 BC, EF and HI loops, respectively; most of the residues at site 2a and site 2b were located in VP1 GH loop and VP2 EF loop, respectively; site 3b was entirely composed of VP2 BC, HI loops and VP3 HI loop. Apart from this, one-third of epitopes were located at N terminus and C terminus (Table 1, Fig. 2). For instance, VP3 N terminus and VP1 C terminus jointly constituted the site 3a in the knob, and VP1 C terminus participated in the construction of site 2b. This demonstrated that although EV-A71 and CV-A16 had 20% differences in amino acid sequence, even 50–60% differences in epitopes, their secondary structures were highly consistent, which made the epitope positions highly consistent. Similarly, the epitope distribution of CV-A10 was highly consistent with that of EV-A71 and CV-A16 due to the highly consistent secondary structures among them (Table 1, Fig. 2).

Superposition of 3D structure of EV-A71 and CV-A16 intuitively showed that the skeletons of structural proteins of them highly overlapped (Supplementary Fig. S2). The difference was measured with the RMSD (C_α_ atoms in a protomer) value of 0.231 Å, revealing the much similar 3D structure shared by those two serotypes (Supplementary Table S1). The RMSDs of three viral proteins (VP1, VP2 and VP3) in EV-A71 and CV-A16 were 0.286 Å, 0.191 Å and 0.191 Å, respectively. It could be seen that the most remarkable structure difference existed in the VP1. Compared with β sheets, there were significant structural variations in the loops, N terminus and C terminus where epitopes occupied owing to the differences in the sequences (Supplementary Fig. S2). Especially, the VP1 BC loop with RMSD up to 0.586 Å. This indicated that the secondary structure and epitope distribution of EV-A71 and CV-A16 were highly consistent, while the difference in the sequence altered the local 3D structure of epitopes, yielding various serotypes. Although the 3D structure of CV-A10 closely resembled that of EV-A71 and CV-A16 (Supplementary Fig. S2), the RMSDs between CV-A10 and EV-A71, CV-A10 and CV-A16 reached 0.466 Å and 0.449 Å (Supplementary Table S1), respectively, exceeding the RMSD between EV-A71 and CV-A16 (0.231 Å). Particularly some loops where epitopes stood, such as VP1 BC loop, HI loop and VP2 EF loop, CV-A10 was quite different from EV-A71 and CV-A16. The surface structures of CV-A10 were more prominent. All of above revealed that EV-A71 and CV-A16 were more similar in amino acid sequences, structures and epitopes compared to CV-A10.

### The reliability of predicted conformational epitopes

**Figure 3:**
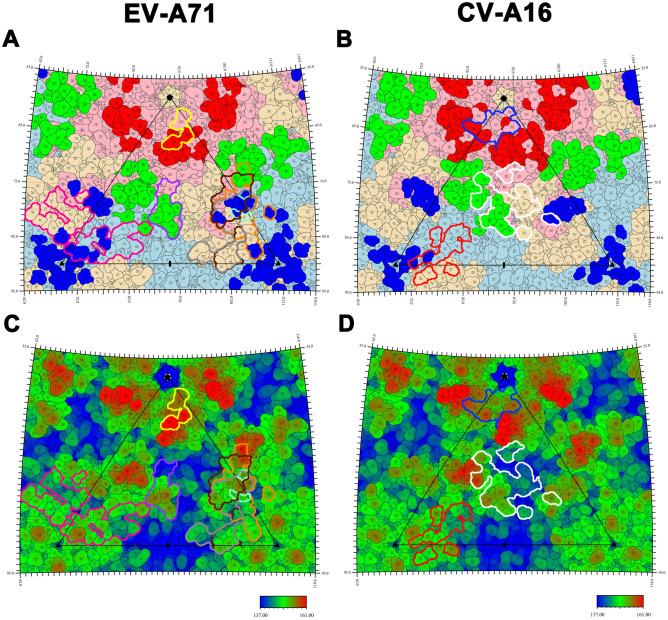
Antibody footprints on the capsid surface of EV-A71 and CV-A16. The capsid surfaces of EV-A71 and CV-A16 are shown as stereographic projections. An icosahedral subunit is outlined by the triangular boundary and the locations of five, three, and twofold vertex are marked as black pentagons, triangles and ovals, respectively. (**A,B**) The residues from VP1 are shown in light pink, VP2 in light blue and VP3 in light orange, respectively; the residues in conformational epitopes site 1, site 2 and site 3 are colored in red, green and blue, respectively. (**A**) The binding sites of seven neutralizing antibodies on EV-A71 are indicated by solid lines in different colors (Mab 28-7: yellow, D5: purple, 10D3: aquamarine, D6: brown, A9: gray, E18: deep pink, E19: orange). (**B**) The binding sites of three antibodies 18A7, 14B10 and NA9D7 on CV-A16 are marked by blue, red and white solid lines, respectively. (**C,D**) The surfaces of EV-A71(**C**) and CV-A16 (**D**) are colored radially from blue through green to red from the lowest to the highest. The binding sites of all antibodies of these two serotypes are also outlined, respecting the color rules in (**A,B**).

Borley et al. had systematically evaluated the reliability of the conformational epitope prediction algorithm^43^. In this study, the reported conformational and linear epitopes of EV-A71 and CV-A16 were obtained through literature retrieval to assess the prediction reliability (Fig. 3, Supplementary Table S2).

At present, seven conformational epitopes of EV-A71 have been reported, mainly including those combined with mAbs 28-7^30^, D5^33^, 10D3^45^, D6^35^, A9^35^, E18 and E19^16^ (Fig. 3A,C). Gradually, three conformational epitopes of CV-A16 also have been unclosed, which were identified by mAbs 18A7, 14B10 and NA9D7, respectively^36^ (Fig. 3B,D). MAb 28–7 bound to site 1 of EV-A71 at the specific binding sites of VP1-98, 145, 242 and 244, of which both 98 and 242 were predicted as conformational epitopes^30^ (Fig. 3A,C). Though VP1-145 and 244 were not predicted, they were adjacent to VP1-242. MAb D5, binding to VP1 GH loop, highly overlapped with site 2a^33^. The specific binding sites of mAb 10D3 were VP3-59, 62, 67 which were located in the knob of site 3a where accommodated the site of mAb 2G8 on CV-A10^37,45^. The binding site of mAb D6, comprised by the VP1 C terminus, VP2 GH loop and puff region of EV-A71, spanned VP1-3 and covered the part of site 2b and entire site 3a. MAb A9 was located at the region between threefold and twofold vertexes, completely covering the site 3a^35^. The epitope which the mAb E18 binding to was close to the threefold vertex and located among site 2, site 3a and site 3b, spanning two neighboring chains and covering partial residues at site 2 and site 3b^31^. The binding sites of mAb E19 were centered on site 3a, covering several or all residues from site 2b, site 3a, and site 3b of two adjacent chains^31^. The binding region of E19 highly overlapped with that of D6 and A9 on EV-A71, suggesting that site 3a is an epitope that readily interacts with antibodies. As regards CV-A16, the mAb 18A7 bound to the BC, DE and HI loops of VP1 and partially overlapped with site 1; the mAb 14B10, binding to the threefold vertex between site 2 and site 3b, highly overlapped with site 3a (Fig. 3B,D)^36^. Specifically, it bound to the BC loop, EF loop of VP3 and some residues from neighboring VP2 BC, EF and HI loops. The combined sites of mAb NA9D7 were located among site 1, site 2 and site 3, covering the canyon and primarily overlapping with site 2. Moreover, the binding sites of 14B10 on CV-A16 were similar to that of E18 on EV-A71 and overlapped with the binding sites of SCARB2^34^. To sum up, each epitope of EV-A71 and CV-A16 could be combined with certain mAbs, and most combination sites of mAbs were centered on three sites (site 1, site 2 and site 3).

Additionally, most of the predicted conformational epitopes were covered with linear epitopes determined by experiments (Supplementary Table S2). For example, the classical epitope SP70 was located in the VP1 GH loop and constituted the site 2a of EV-A71^46^; SP32 binding to the VP1 BC loop and SP55 to VP1 EF loop constituted site 1a and site 1b, respectively^46,47^; VP2-28 lying in the VP2 EF loop formed the site 2b^48^. Similarly, the linear epitopes of CV-A16 also overlapped with the conformational epitopes. PEP32 was located in the VP1 BC loop and constituted the site 1a of CV-A16; PEP71, sitting in the VP1 GH loop, constituted site 2a^49^; VP3B was located at the N terminus of VP3 and become a part of site 3a^50^. Furthermore, it could be seen that each conformational epitope of CV-A16 and EV-A71 was covered by linear epitope excepting the site 3 of EV-A71 (Supplementary Table S2). This indicated that our predicted results of conformational epitopes were reliable and the epitopes were probably the key parts of linear epitopes, which could provide important guidance for efficiently designing linear epitopes.

As the representative serotype of EV-C, PV1 owned the highly consistent epitope distribution pattern (three-site distribution) with EV-A (EV-A71, CV-A16 and CV-A10) (Supplementary Fig. S3, S4). Of particular interest, the site 1, site 2 and site 3 exactly corresponded to the three well-known epitopes (N-AgI, N-AgII and N-AgIII) of PV1, respectively (Supplementary Fig. S5). This fully validated the reliability of prediction algorithm.

### The relationship between conformational epitopes and receptor binding sites

**Figure 4:**
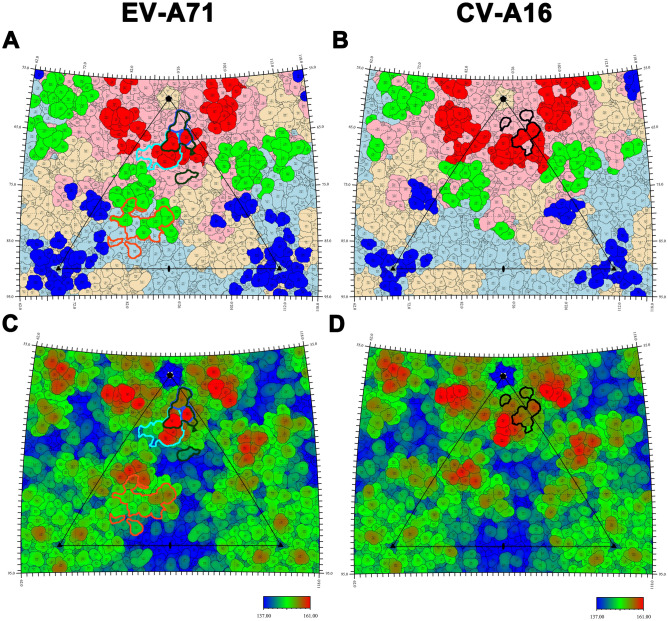
Receptor footprints on the capsid surface of EV-A71 and CV-A16. The icosahedral subunit and color role are the same as Fig. 3. The footprints of different receptors are indicated by colorful solid lines. (**A**,**C**) The footprints of four receptors SCARB2, PSGL-1, HS and Anx2 on EV-A71 are circled in dark orange, blue, dark green, cyan, respectively. (**B,D**) The footprint of HS on CV-A16 is circled by black.

The binding of receptor to virus was the first and crucial step for virus infection. Currently, six cell receptors of EV-A71 have been found, namely Scavenger receptor class B member 2 (SCARB2)^34^, P-selectin glycoprotein ligand-1 (PSGL-1)^51^, Heparan sulfate (HS)^52^, Annexin II (Anx2)^53^, Sialylated glycans^54^ and Dendritic cell-specific ICAM3-grabbing non-integrin (DC-SIGN)^55^. Among them, SCARB2 was an uncoating receptor of EV-A71, while the other five were attachment receptors that supported viral attachment to the cell surface and failed in triggering uncoating. Luckily, the binding sites of SCARB2, PSGL-1, HS and Anx2 have been clearly identified, while that of Sialylated glycans and DC-SIG remained unknown (Fig. 4A,C). Of note, HS was also the attachment receptor of CV-A16 (Fig. 4B,D)^56^. Moreover, SCARB2 and PSGL-1 can also bind to CV-A16, but the specific binding sites have not been determined yet.

Previously, it was believed that SCARB2 possibly bound to both rims of the canyon, but the latest research revealed that SCARB2 bound to the puff on the southern rim^34,57^. It interacted with VP1 GH loop and VP2 EF loop which constituted site 2a and site 2b, respectively (Fig. 4A,C). MAb D5 bound to the VP1 GH loop and partially overlapped with the binding site (VP1-216, 217) of SCARB2 (Figs. 3A, 4A). The epitopes which the mAb E18 binding to were near the threefold vertex and overlapped with the binding sites (VP2-78, 149, 156, 158) of SCARB2 (Figs. 3A, 4A). Among them, VP1-216, 217 and VP2-149 were crucial residues of site 2. MAb E18 can initiate EV-A71 to convert from mature particle to A-particle and release the genome. The binding sites of mAb 14B10 and NA9D7 to CV-A16 also overlapped with the site of SCARB2 (Figs. 3B, 4A). Nevertheless, it was still unclear whether their neutralization mechanisms were related to SCARB2.

The binding sites of PSGL-1, HS and Anx2 to EV-A71 were located in the site 1 or nearby (Figs. 3A, 4A). Nishimura et al. found that VP1-145, 242, 244 were the key residues affecting EV-A71 binding to PSGL-1^51^. Among them, VP1-242 was a member of site 1c, while 145 and 244 were near site 1c. The binding site of HS highly overlapped with that of PSGL-1. Except for VP1-145, 242 and 244, the adjacent VP1-97, 98, 162 and 166 were also the binding sites of HS, which highly overlapped with site 1. Compared with EV-A71, the binding sites of HS to CV-A16 were slightly different, specifically VP1-141, 166, 241, 242 and 245, which also highly overlapped with the site 1c (Figs. 3B, 4B)^56^. The binding site of Anx2 to EV-A71 is VP1-40–100, partially overlapped with the epitope site 1a (Figs. 3A, 4A)^53^. For EV-A71, the binding sites of mAb 28-7 highly overlapped with that of PSGL-1 and HS and partially overlapped with that of Anx2. For CV-A16, the binding sites of mAb 18A7 overlapped the part of HS binding sites. This elucidated that neutralizing antibodies may competitively bind to these epitopes with host cell receptors.

### Intra-serotype genetic diversity analysis

Intra-serotype genetic diversity analysis was performed to explore whether high-frequency mutations occurred in epitopes since enteroviruses evolved quickly. Totally, there were 1224, 221, 177 and 264 complete genome sequences available from NCBI Nucleotide database for EV-A71, CV-A16, CV-A10 and PV1, respectively. It could be clearly observed that several residue positions of EV-A71 loops and C terminus involved in epitope regions had high diversities (Supplementary Fig. S6, Table 1), though few residues of them (such as VP1-98 and VP2-144) were predicted as epitopes directly. For example, C terminus of VP3 had high diversity, but only VP3-240 was part of site 2. This implied that current mutations of EV-A71 may not affect the protection of vaccines but be worth continuous surveillance. On the contrary, few residue positions of CV-A16 had high diversity except for some in VP1 and VP2 HI loops, so CV-A16 was considered as a highly conserved serotype. Interestingly, two serotypes CV-A10 (EV-A) and PV1 (EV-C) both had high diversities like EV-A71 and most diverse positions occurred in epitope regions. According to PV1, BC and GH loops of VP1, EF loop of VP2 and N terminus of VP3, which were key regions of N-AgI, N-AgII and N-AgIII (Supplementary Fig. S5, S6), all exhibited great diversity. The intra-serotype genetic diversity analysis hinted that variation surveillance for epitopes was of great importance.

## Discussion

Determination of antigenic epitopes, especially the conformational epitopes, is critical for the development of diagnostic reagents and supervision of the virus mutation. As to influenza viruses, the surveillance and analysis of antigenic epitopes have proceeded for several years, which can help to predict the transformation of virus antigenicity, develop and prepare the vaccines^24–28^. The prediction of conformational epitope belongs to the traditional field of Bioinformatics research. The classical conformational epitope prediction algorithms such as Ellipro, DiscoTope, and Epitopia all regard proteins as spheres or ellipsoids and predict the most prominent part of the surface that is most easily bound to antibodies as the conformational epitope^40–42^. Different from the HA and NA of influenza viruses, the structural proteins of human enterovirus do not exist and function independently but are assembled into a capsid to perform their biological functions^17,58^. As a result, most of the residues in the structural proteins are not exposed on the capsid surface and cannot act as conformational epitopes. If this property is not considered, the classical algorithm such as DiscoTope may incorrectly predict a large number of non-exposed amino acid residues as conformational epitopes^40^. Borley et al. developed a Bioinformatics-based algorithm for predicting the conformational epitopes of FMDV, showing high prediction reliability^43^. The pivotal concept of this algorithm is that, as the state of chain 1 in the multiple chains is exactly the same as its state on the capsid, the prediction results of the central chain should be theoretically correct. Both enterovirus and FMDV belong to the family *Picornaviridae* and have similar capsid structures. Therefore, we developed an algorithm for predicting the conformational epitopes of human enterovirus based on Borley et al.’s algorithm. This algorithm has been used to successfully predict the epitopes of CV-A10 and proved to be highly reliable^44^. And the prediction result showed that the conformational epitopes of CV-A10 were clustered into three sites (site 1, site 2 and site 3). In this study, we predicted the conformational epitopes of EV-A71 and CV-A16 and conducted the comparison with that of CV-A10. The final predicted epitopes consisted of core epitopes and surrounding epitopes, with the core epitopes defined by a rigid standard that they were simultaneously predicted via Ellipro, DiscoTope, and Epitopia. Nevertheless, the surrounding epitopes just needed to be predicted as epitopes by any two prediction tools of Ellipro, DiscoTope and Epitopia, and were the core epitopes of other related serotypes of enterovirus. For example, the 94D in VP1 of EV-A71 was predicted by two tools; meanwhile, it was also the core epitope of CV-A16. Thus, it was defined as surrounding epitope. Such algorithm design is based on the hypothesis of “similar structure, similar epitope”, with considering that the resolutions of 3D structures are not completely the same and some random errors occur in determination of structures. This kind of algorithm design will not increase the number of epitopes but enhance the exhibition of epitope distribution pattern.

EV-A71, CV-A16, CV-A10 and CV-A6 are the predominately causative agents of HFMD. Among them, EV-A71 and CV-A16 were the most common pathogens in the early outbreaks of the Asia–Pacific region. However, CV-A10 and CV-A6 have become the dominant agents and responsible for several outbreaks of HFMD in recent years^10,59^. Vaccines for various viral diseases can reduce mortality and morbidity worldwide. Friendly, determination of epitopes of enteroviruses can be helpful to monitor their mutations and develop vaccines. Because of the 3D structure of CV-A6 mature virion not identified, our algorithm based on the structure wasn’t permitted. In the present study, we systematically predicted the conformational epitopes of EV-A71 and CV-A16 and compared with our previous study (CV-A10)^44^. The result revealed that the conformational epitopes of EV-A71, CV-A16 and CV-A10 had the same distribution pattern. Specifically, all epitopes consisted of three clusters: site 1, site 2 and site 3. Site 1 was located on the northern rim of the canyon near the fivefold vertex, and site 2 in the puff. Site 3 was divided into two parts: one was located in the knob, and the other was close to the threefold vertex. As representative enteroviruses, PV1 and RVB14, their antigenic epitopes have been systematically explored and epitope-distribution patterns have been summarized^39,60^. The epitopes of PV1 and RVB14 were both clustered into three sites (PV1: N-AgI, N-AgII and N-AgIII; RVB14: NIm-I, NIm-II and NIm-III) and these sites corresponded to three sites of EV-A71 and CV-A16^39,60^. Overall, these data implied the prediction results were of great reliability and three-site distribution pattern was the fundamental role of the conformational epitope distribution of enterovirus.

Fivefold vertex (site 1), puff (site 2), and knob (site 3) are important characteristics of the enterovirus capsid and most easy to bind to mAbs and receptors. The VP1 loops of five asymmetric subunits are arranged in a crater-like structure, making the area around fivefold vertex most prominent on the capsid surface. MAbs 28-7 (EV-A71)^30^, 18A7 (CV-A16)^36^, 1D5 (CV-A6)^61^ and 11G1 (EV-D68)^62^ all interacted with this area where could also accommodate the attachment receptors PSGL-1^51^, HS^52^, and Anx2^53^ of EV-A71. The binding of mAbs or receptors to the virus might be regulated by epitopes or nearby residues. For example, the VP1-145, near the epitope residue VP1-242, could regulate the binding of PSGL-1 to EV-A71. When the 145 mutated from G/Q to E, a negatively charged region was formed. It reduced the prominence of the positively charged 242 K side chain and eliminated the ability of EV-A71 binding to PSGL-1^51^. Analogously, both mAbs 28–7 (EV-A71) and 18A7 (CV-A16) can only bind to 145G rather than 145E^30,36^. Puff region, the most protruding structure of capsid second to the fivefold vertex, mainly consisted of VP1 GH loop and VP2 EF loop which accommodated the linear epitopes SP70 and VP2-28, respectively^46,48^. As respected, mAbs D5 and NA9D7 combined with this region as well and the location of mAb E18 (EV-A71) likewise overlapped with it^31,33,36^. In addition, the uncoating receptor SCARB2 partially bound to this region. Unluckily, it was still unknown whether the uncoating mechanism of E18 was consistent with SCARB2^34^. Knob region was another protrusion on the capsid surface which interacted with mAbs 10D3, D6, A9, E19 (EV-A71) and 2G8 (CV-A10)^37^. Also, the binding sites of NA9D7 (CV-A16) involved some residues in the knob. Unlike the binding sites of SCARB2 on EV-A71, the KREMEN1 on CV-A10 covered the entire canyon and contacted with the fivefold vertex, puff and knob region^63^. Of note, there were some receptor-specific amino acid residues in the EF loop of VP2. For instance, the K140 and P141 of VP2 which the KERMEN1 bound to were conserved in multiple enteroviruses including CV-A10, while the residue on the same position of EV-A71 and CV-A16 was G140 and T/N141, respectively. Conversely, the G137 of VP2 was a conserved residue for SCARB2 binding to in multiple enteroviruses including EV-A71, while the S137 was in the CV-A10. This suggested that the residues in the epitopes were essential for receptor-specific binding.

Intra-serotype genetic diversity analysis revealed that BC and HI loops, and C terminus of VP1, EF and HI loops of VP2, and C terminus of VP3 were the regions with high diversity, which exhibited that these secondary structures involved in epitopes were susceptible to high-frequency mutations. Since only a few residue positions of epitopes had high diversity (for example, VP1-98 and VP2-144 of EV-A71) and most residues of epitopes were conserved, the genetic divergence such as genotypes/clades and the genetic diversity did not alter the antigenicity significantly till now. The conservation of epitopes was consistent with the results of cross-reactions. On the one hand, EV-A71 vaccine and most antibodies/antisera could provide cross-protection against strains of various genotypes^64–66^. On the other hand, a handful of antibodies/antisera could only cross-react with specific genotype strains, which illustrated that both EV-A71 and CV-A16 had relatively stable antigenicity, but some virus strains might have antigenic variations due to mutations in the epitopes^67,68^. Thus, it was important to surveil the variations in epitopes continuously.

Our Bioinformatics epitope prediction method still has some defects. Firstly, this is a structure-based prediction algorithm, and if the 3D structures of virus particles (such as CV-A6 mature virus particles) are not determined, the algorithm will be denied. Secondly, some missing structures in the 3D structures (such as disordered loops) will affect the prediction. Finally, the object of the algorithm is to find the residues on the surface of viral capsid that are easy to bind to antibodies or receptors. However, the bottom of the canyon, a special area, bound by certain receptors is out of consideration.

In summary, our conformational epitope prediction results suggest that EV-A such as EV-A71, CV-A16 and CV-A10 have a similar three-site distribution pattern. And sequence variations lead to noticeable 3D structural differences in the loops, N terminus and C terminus where the epitopes reside. This may yield different serotypes that cannot be cross-protected by vaccines. Our research shows that Bioinformatics prediction results can provide meaningful information for epitope research and identification, linear epitope design and important targets for virus mutation surveillance. In the future, we will use these epitopes to study and predict the antigenic evolution and epidemic trends of the major pathogens of HFMD to help monitor its outbreak.

## Materials and methods

### Sequence and structure analysis

**Figure 5:**
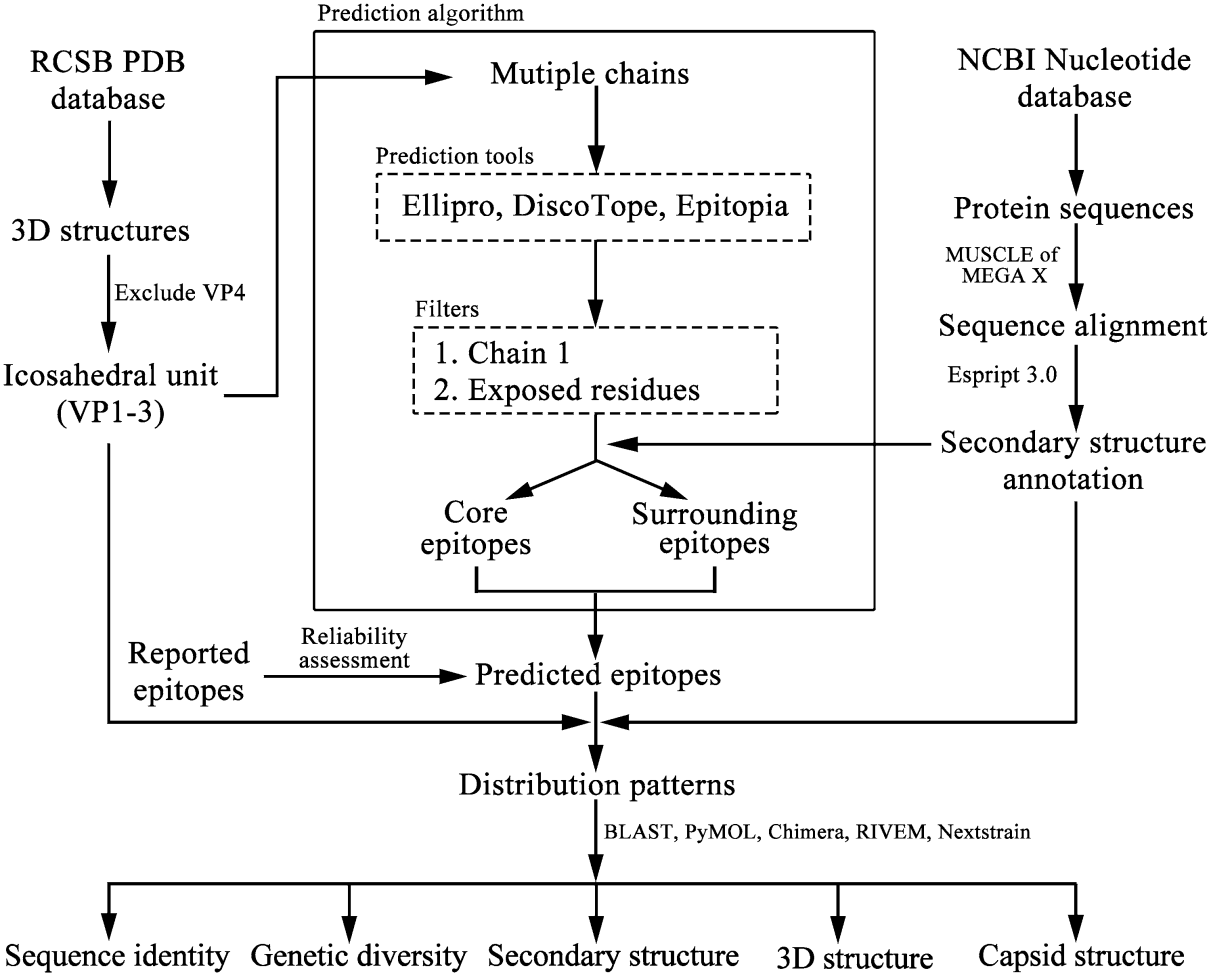
Analysis workflow.

EV71/Fuyang.Anhui.P.R.C/17.08/1 (GenBank accession no: EU703812) and Ningbo.CHN/028-2/2009 (GenBank accession no: JQ354992) strains, whose 3D crystal structures have been determined, were selected as representative strains of EV-A71 and CV-A16, respectively. EV-A71 strain, isolated in Fuyang City, Anhui Province, China in 2008, belongs to subgenotype C4. And CV-A16 strain, isolated in Ningbo City, Zhejiang Province, China in 2009, belongs to subgenotype B1a. The 3D structures of EV-A71 mature virus (PDB ID: 3VBS) and CV-A16 mature virus (PDB ID: 5C4W) were obtained from the RCSB Protein Data Bank (PDB) database (http://www.rcsb.org)^69^. The deduced protein sequences were downloaded from the National Center for Biotechnology Information (NCBI) Nucleotide database (https://www.ncbi.nlm.nih.gov/nuccore/). Also, the 3D structure (PDB ID: 6AKS) and protein sequence (GenBank accession no: KP009574) of CV-A10 (strain FY01/AH/CHN/2013) were downloaded to conduct the sequence and structure comparison. The protein sequences of EV-A71, CV-A16 and CV-A10 were aligned by MAGE X using MUSCLE^70^, then uploaded with corresponding PDB files to the ESPript 3.0 (http://espript.ibcp.fr/ESPript/cgi-bin/ESPript.cgi) for noting secondary structures information^71^. The sequence alignment was uploaded to BLAST (https://blast.ncbi.nlm.nih.gov/Blast.cgi) for calculating the amino acid residue identity. The 3D structures of viral proteins and capsid surfaces were generated and compared by UCSF Chimera^72^ and PyMOL^73^. The root mean square deviation (RMSD) of structural difference was calculated by PyMOL. All of software set the default parameters. To perform intra-serotype genetic diversity analysis, all complete genome sequences of EV-A71, CV-A16, CV-A10 and PV1 (used for reliability assessment) were downloaded from NCBI Nucleotide database and the deduced protein sequences for VP1-3 were truncated. Entropy based on amino acid residues was calculated to perform diversity analysis using Augur package of Nextstrain platform^74^. The whole analysis workflow was presented in Fig. 5.

### Bioinformatics-based prediction of conformational epitopes

Borley et al. developed a Bioinformatics-based algorithm to predict the conformational epitopes of FMDV by generating the multiple chains^43^. Based on that, we developed a Bioinformatics algorithm to predict the conformational epitopes of human enteroviruses and successfully applied it on CV-A10^44^. The prediction steps of the algorithm were as follows (Fig. 5):

Firstly, construct the multiple chains of EV-A71 and CV-A16 (Fig. 1D,E,G,H). The information of VP4 was deleted and VP1-3 were kept in PDB file as the central chain 1. The central chain 1 and surrounding chains (chain 2/3/4) were generated by epiprep based on 3D coordinates and non-crystallographic symmetry (NCS)^43^. The same method was employed to build a whole viral capsid (excluding VP4) containing 60 chains (Fig. 1A,B).

Secondly, predict the epitopes of EV-A71 and CV-A16. Three classic prediction tools ElliPro (http://tools.iedb.org/ellipro)^41^, DiscoTope (http://www.cbs.dtu.dk/services/DiscoTope)^40^ and Epitopia (http://epitopia.tau.ac.il/index.html)^42^ were employed to predict the multiple chains and the thresholds were set 0.3, -10.7 and 0.174, respectively^43^. Residues on VPs were predicted as candidate epitopes for whose prediction values were not less than thresholds.

Thirdly, screen the prediction results of multiple chains. First, the prediction result of chain 1 was selected. Viral capsid consisted of sixty chains, one of which was surrounded by three other chains and the interfaces between them were not exposed on the surface to form epitopes (Fig. 1A–C). So, as for multiple chains, only central chain 1 was totally surrounded by other chains, and its location was the same as its on the capsid (Fig. 1D–I). Then, amino acid residues exposed on the outer surface (exposed residues) of capsid were selected. Concretely, the average distance between C_α_ of each residue to capsid center was calculated and those whose distances were over the average were deemed as exposed residues.

Finally, determine the final conformational epitopes by a voting method. Those residues, simultaneously predicted by all three prediction tools (ElliPro, DiscoTope and Epitopia), were defined as core epitopes. Additionally, the residues were merely predicted by any two tools whilst be predicted as core epitopes by other enteroviruses, can be defined as surrounding epitopes. Ultimately, the predicted epitopes included the core epitopes and surrounding epitopes.

### The reliability of predicted conformational epitopes

The binding sites of receptors and antigenic epitopes identified by experiments (experimental epitopes) were obtained from reported researches. Predicted epitopes and experimental epitopes were compared to evaluate the reliability of algorithm. The viral capsid “footprint” of EV-A71 and CV-A16 were produced by RIVEM^75^. Predicted epitopes, experimental epitopes and receptor-binding sites were marked on footprints to analyze the relationship among them. PV1 is the representative serotype of EV-C and the antigenic epitopes have been extensively and systematically studied. Therefore, we also predicted the conformational epitopes of PV1 (PDB ID: 1HXS) to help assess the prediction reliability.

## Supplementary Information

Supplementary information.
