# Supplementary material for: Bioinformatics-based prediction of conformational epitopes for Enterovirus A71 and Coxsackievirus A16
Source: Sci Rep. 2021 Mar 11;11:5701. doi: 10.1038/s41598-021-84891-6 (PMC7952546; doi:10.1038/s41598-021-84891-6)
Supplement: Supplementary file 1 — Supplementary information. [file 41598_2021_84891_MOESM1_ESM.pdf]

Supplementary Material

Bioinformatics-based Prediction of Conformational Epitopes for Enterovirus A71 and Coxsackievirus A16

Liping Wang[1,+], Miao Zhu[1,2,+], Yulu Fang[1], Hao Rong[1], Liuying Gao[1,3], Qi Liao[1], Lina Zhang[1], Changzheng Dong[1,*]

[1] Department of Preventive Medicine, Zhejiang Provincial Key Laboratory of Pathological and Physiological Technology, School of Medicine, Ningbo University, Ningbo, China

[2] Department of Infection Control, Heze Municipal Hospital, Shandong, China

[3] The Affiliated People's Hospital of Ningbo University, Ningbo, China

[*]dongchangzheng@nbu.edu.cn

[+]these authors contributed equally to this work

Supplementary Table S1 The RMSD of viral proteins of EV-A71, CV-A16 and CV-A10.

Viral protein	Secondary structure	RMSD (Å)		
		EV-A71/CV-A16	EV-A71/CV-A10	CV-A16/CV-A10
VP1-3	-	0.231	0.466	0.449
VP1	-	0.286	0.517	0.487
	BC loop	0.586	0.671	0.938
	EF loop	0.185	0.320	0.262
	GH loop	0.204	0.248	0.354
	HI loop	0.355	0.688	0.601
	C terminus	0.217	0.501	0.763
VP2	-	0.191	0.372	0.360
	BC loop	0.152	0.342	0.369
	EF loop	0.150	0.373	0.394
	HI loop	0.133	0.301	0.323
VP3	-	0.191	0.448	0.395
	N terminus	0.183	0.634	0.808
	HI loop	0.115	0.367	0.400
	C terminus	0.122	1.093	1.080

Supplementary Table S2 The linear epitopes of EV-A71 and CV-A16.

Serotype	Epitope	Name of linear epitope	Position of linear epitope
EV-A71	site 1a	SP32	VP1-97-105
	site 1b	SP55	VP1-163-177
	site 1c	4E8	VP1-240-260
	site 2a	SP70	VP1-208-222
		VP1-43	VP1-211-220
	site 2b	VP2-28	VP2-136-150
		VP2	VP2-141-155
		7C7	VP2-142-146
CV-A16	site 1a	PEP32	VP1-94-108
		VP1E	VP1-96-105
	site 1b	VP1F	VP1-159-169
		PEP55	VP1-163-177
	site 1c	VP1I	VP1-238-245
	site 2a	PEP71	VP1-211-225
		VP1H	VP1-212-224
	site 2b	PEP91	VP1-271-285
		VP1K	VP1-272-282
		VP2C	VP2-139-153
	site 2c	VP3E	VP3-229-237
	site 3a	VP1L	VP1-287-295
		VP3B	VP3-56-66
	site 3b	VP2D	VP2-222-230

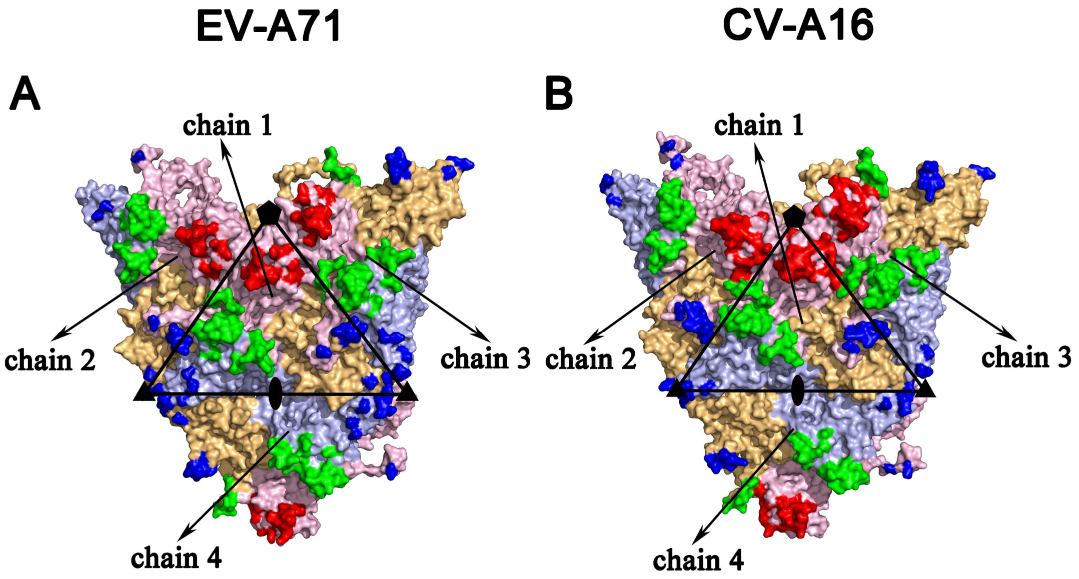

Supplementary Fig. S1 Predicted conformational epitopes on multiple chains of EV-A71 and CV-A16.

The complete conformational epitopes on multiple chains of EV-A71 (A) and CV-A16 (B) are displayed. Viral proteins VP1-3 are colored in light pink, light blue and light orange, respectively; residues involved in epitopes site 1, site 2 and site 3 are shown in red, green and blue, respectively. The icosahedral asymmetric units are outlined by big black triangles. Pentagons, small triangles and ovals represent the fivefold, threefold, and twofold vertexes.

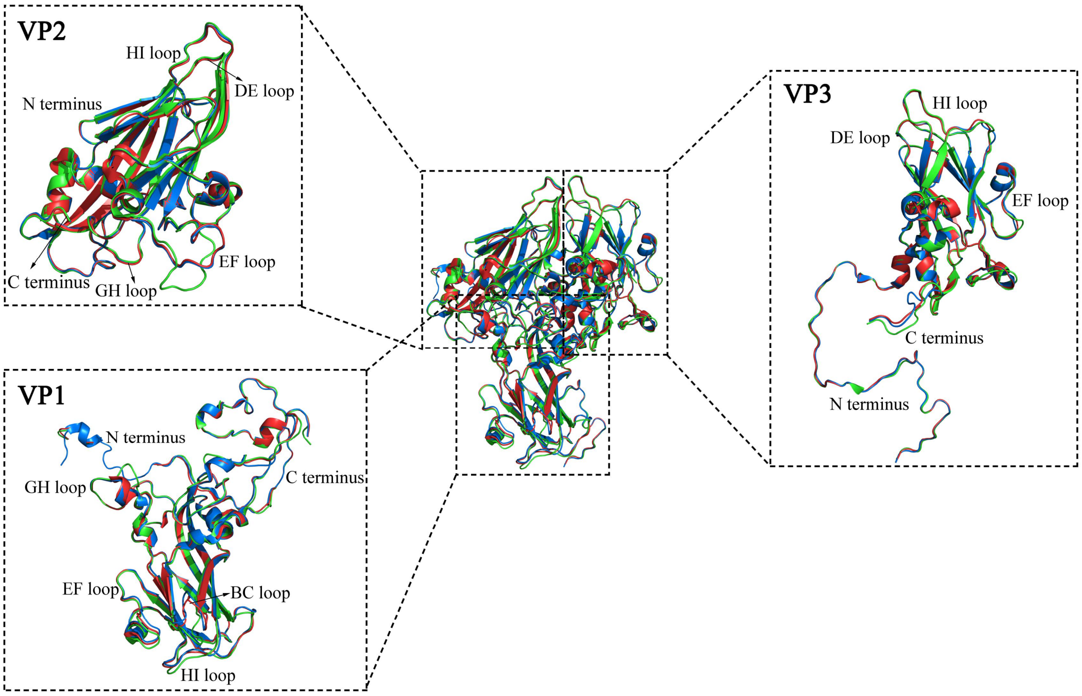

Supplementary Fig. S2 Comparison of β-barrels in EV-A71, CV-A16 and CV-A10.

Superimpositions of icosahedral asymmetric units of EV-A71 (marine), CV-A16 (red) and CV-A10 (green) are displayed. Zoom-in views of VPs are shown in dashed frames. N terminus, C terminus and some loops are already labeled.

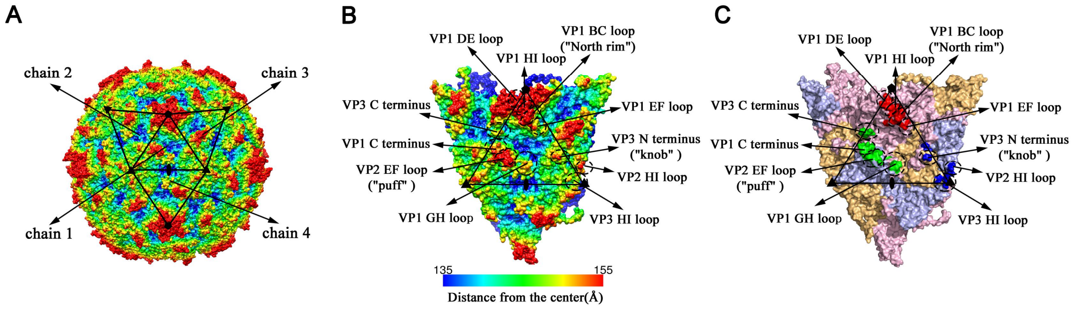

Supplementary Fig. S3 Capsid structures of PV1.

The icosahedral subunit and color scheme are the same as Fig 1.

VP1

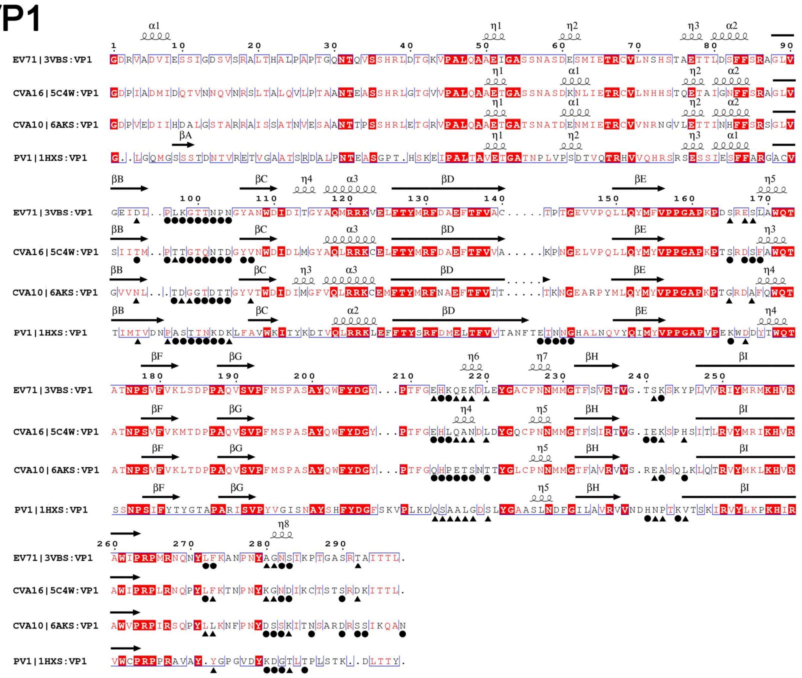

VP2

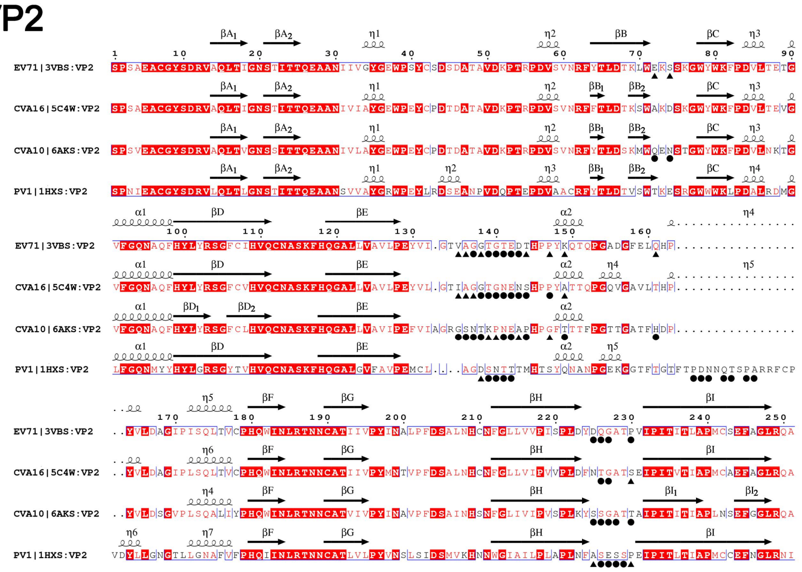

VP3

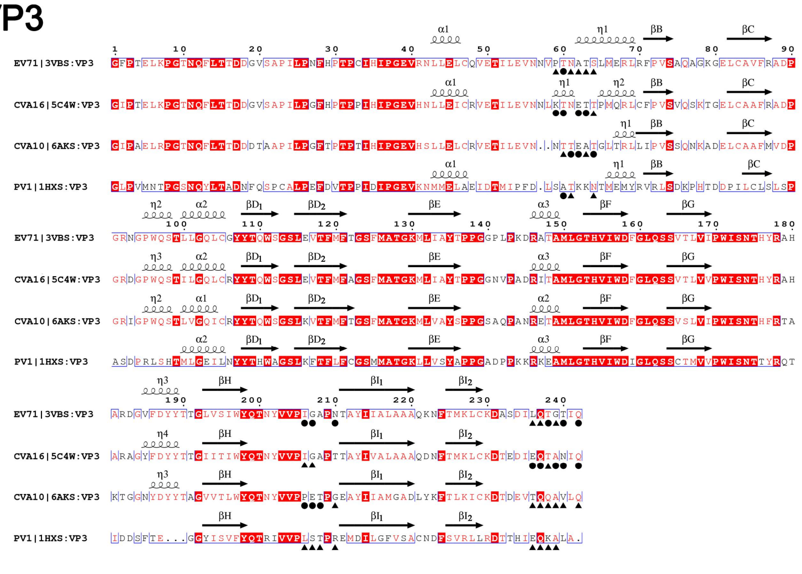

Supplementary Fig. S4 Sequence alignments of three VPs (VP1-3) of EV-A71, CV-A16, CV-A10 and PV1.

The secondary structure elements and symbols are the same as Fig 2.

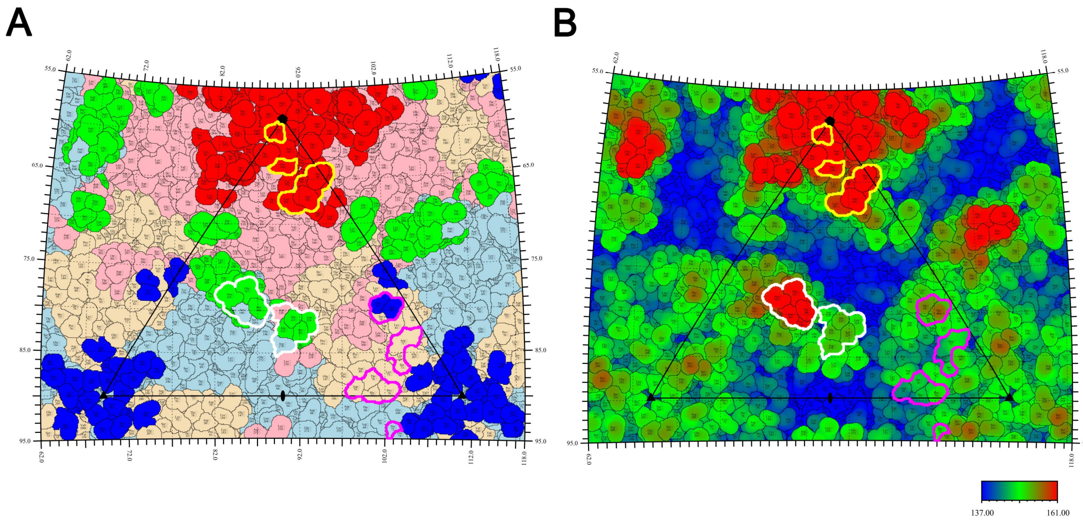

Supplementary Fig. S5 Footprints of reported epitopes on the capsid surface of PV1.

The icosahedral subunit and color scheme are the same as Fig 3. The well-known epitopes N-AgI, N-AgII and N-AgIII are circled by yellow, white and magenta, respectively.

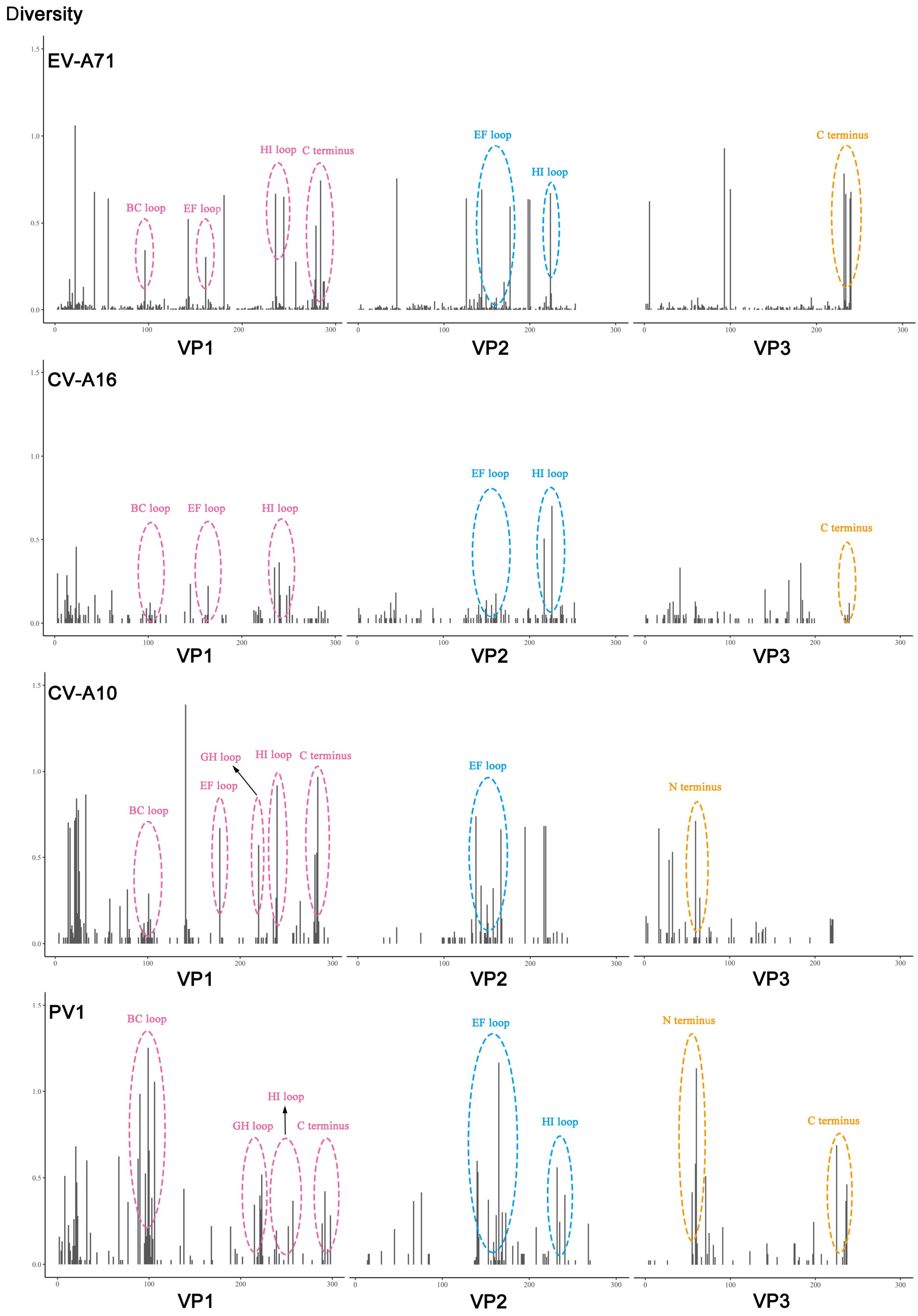

Supplementary Fig. S6 The amino acid residue diversity on VPs of EV-A71, CV-A16, CV-A10 and PV1.

The major epitope regions with high diversity on VPs were outlined.
